# A Man With Neck Pain and Swelling

**DOI:** 10.1016/j.acepjo.2026.100408

**Published:** 2026-04-29

**Authors:** Joshua M. Perez-Cruet, Summer Ghaith, Bryan Cortez, John L. Kendall, Timothy J. Batchelor

**Affiliations:** Department of Emergency Medicine, Division of Emergency Ultrasound, Stanford University, Palo Alto, California, USA

**Keywords:** ultrasound, POCUS, ultrasonography, Ludwig's angina, abscess

## 1 Patient Presentation

**Figure 1:**
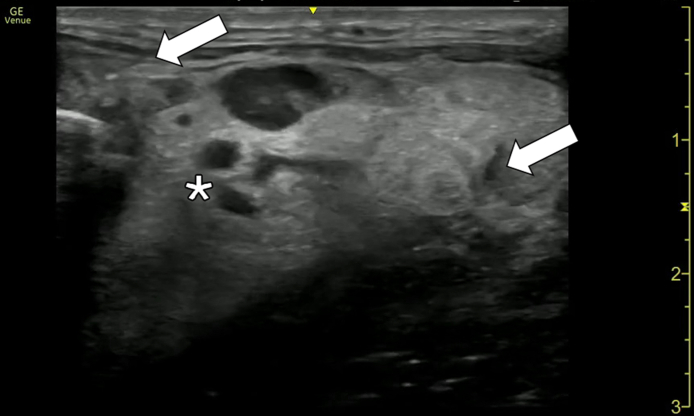
Transverse point-of-care ultrasound image of the submandibular neck revealing cobblestoning with subcutaneous fluid collection (arrows), lymphadenopathy, and loculated fluid collections along the left mandible (star).

**Figure 2:**
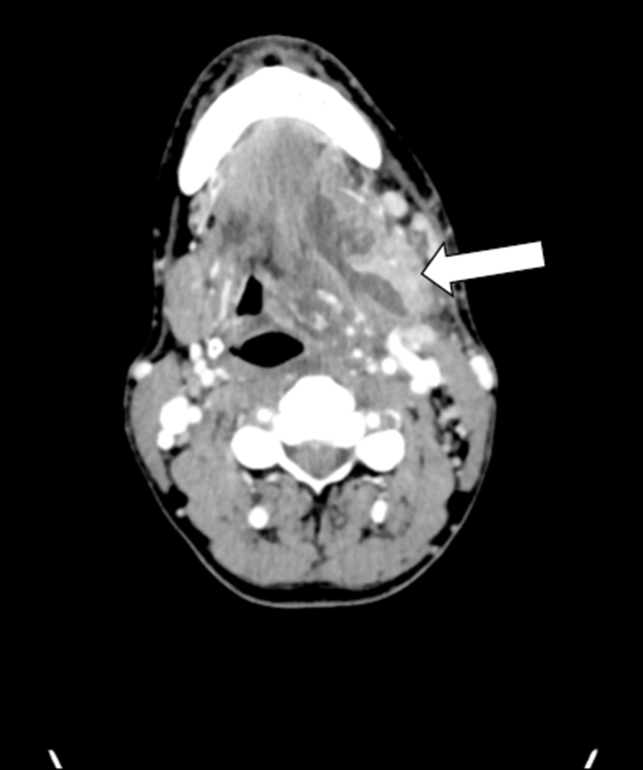
Axial contrast-enhanced computed tomography of the neck revealing a 4.7 cm multiloculated fluid collection (arrow) within the left submandibular space, with surrounding edema, inflammation, and gas collection.

**Figure 3:**
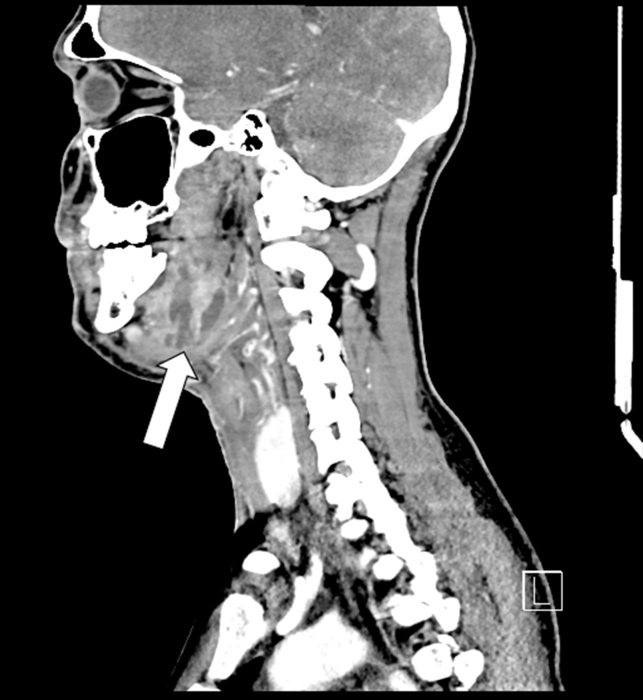
Coronal contrast-enhanced computed tomography of the neck revealing a 4.7 cm multiloculated fluid collection (arrow) within the left submandibular space, with surrounding edema, inflammation, and gas collection.

A 47-year-old man presented to the emergency department with neck pain and swelling. Symptoms began 1 week prior to arrival with difficulty swallowing liquids, and on arrival, he endorsed increased swelling, tongue elevation, and difficulty breathing while supine. He was afebrile, and SPo_2_ was above 95% on room air. Examination was notable for a firm, tender 2-cm left submandibular mass with surrounding edema and erythema, mouth floor edema, elevated tongue, rightward tracheal displacement, and trismus. Point-of-care ultrasound (POCUS) of the neck was performed (Fig 1, Video 1) followed by a computed tomography (CT) (Figs 2 and 3).

**Video 1:**
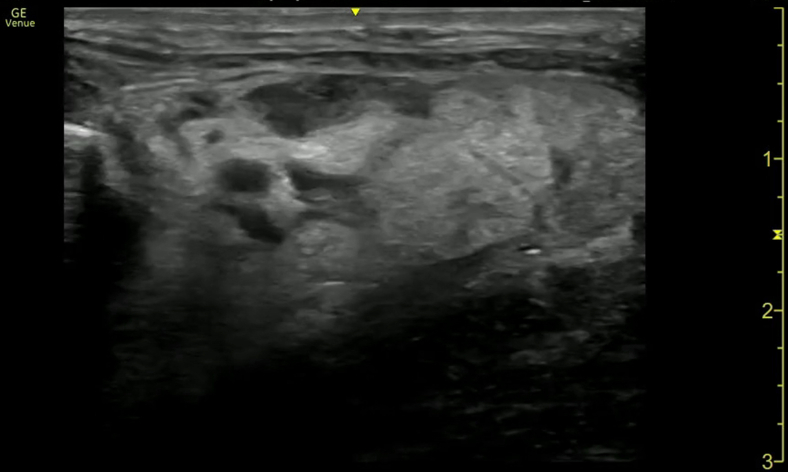
Point-of-care ultrasound with a linear probe showing the transverse view of the submandibular neck. There is cobblestoning of the subcutaneous tissue with subcutaneous fluid collections, an enlarged lymph node, and sonographic evidence of abscess.

## 2 Diagnosis: Ludwig’s Angina

*Ludwig’s* *angina* is a rare, aggressive cellulitis involving the submandibular space, which can rapidly compromise the airway.^1^ Traditional risk factors include oropharyngeal infection (most commonly a mandibular molar), recent upper respiratory infection or oral procedure, immunodeficiency, and substance use.^1^ Typically, cultures identify polymicrobial oral anaerobes, but immunocompromised patients are at increased risk of infection with gram negative anaerobes and methicillin-resistant *Staphylococcus aureus*.^1^^,^^2^ Although preferred imaging is contrast-enhanced CT, POCUS may be used if patients cannot tolerate lying supine or are too unstable for CT imaging, and can provide accelerated time to diagnosis and treatment.^1^ POCUS can help differentiate between superficial and deeper neck space infections or delineate neck anatomy prior to a surgical airway.^3^^,^^4^ Treatment is with airway management, broad spectrum antibiotics, source control, and intensive care unit admission for airway monitoring.^1^^,^^5^ Signs of airway compromise should prompt rapid intervention, ideally with the use of awake fiberoptic nasal intubation and surgical airway as an immediate back-up.^1^^,^^6^

## Funding and Support

By *JACEP Open* policy, all authors are required to disclose any and all commercial, financial, and other relationships in any way related to the subject of this article as per ICMJE conflict of interest guidelines (see www.icmje.org). The authors have stated that no such relationships exist.

## Conflict of Interest

We wish to confirm that there are no known conflicts of interest associated with this publication and there has been no significant financial support for this work that could have influenced its outcome. We confirm that the manuscript has been read and approved by all named authors and that there are no other persons who satisfied the criteria for authorship but are not listed. We further confirm that the order of authors listed in the manuscript has been approved by all of us. We confirm that we have given due consideration to the protection of intellectual property associated with this work and that there are no impediments to publication, including the timing of publication, with respect to intellectual property. In so doing we confirm that we have followed the regulations of our institutions concerning intellectual property and patient confidentiality. We understand that the Corresponding Author is the sole contact for the Editorial process (including Editorial Manager and direct communications with the office). He is responsible for communicating with the other authors about progress, submissions of revisions and final approval of proofs. We confirm that we have provided a current, correct email address which is accessible by the Corresponding Author and which has been configured to accept email at jperezcr@stanford.edu.
